# Circulating MiRNA-195-5p and -451a in Transient and Acute Ischemic Stroke Patients in an Emergency Department

**DOI:** 10.3390/jcm8020130

**Published:** 2019-01-22

**Authors:** Mauro Giordano, Tiziana Ciarambino, Michele D’Amico, Maria Consiglia Trotta, Alessandra Marinella Di Sette, Raffaele Marfella, Lorenzo Malatino, Giuseppe Paolisso, Luigi Elio Adinolfi

**Affiliations:** 1 Department of Advanced Medical and Surgical Sciences, University of Campania “L. Vanvitelli”, 80138 Naples, Italy; Tiziana.ciarambino@gmail.com (T.C.); alessandra.marinella@inwind.it (A.M.D.); raffaele.marfella@unicampania.it (R.M.); giuseppe.paolisso@unicampania.it (G.P.), luigielio.adinolfi@unicampania.it (L.E.A.); 2 Department of Experimental Medicine, Division of Pharmacology, University of Campania “L. Vanvitelli”, 80138 Naples, Italy; michele.damico@unicampania.it (M.D.),mariaconsiglia.trotta2@unicampania.it (M.C.T.); 3 Department of Medicine, Section of Hypertension and Cardio-Renal Diseases, University of Catania, 95126 Catania, Italy; malatino@unict.it

**Keywords:** miRNA, acute ischemic stroke, transient ischemic attack, emergency

## Abstract

We have evaluated circulating miRNAs (-195-5p and -451a) in subjects with acute ischemic stroke (AIS) and in patients with transient ischemic attack (TIA). In this study, 18 subjects with AIS and 18 patients with TIA were enrolled and examined at admission (T0) and at 24 h and 48 h after admission, and compared to 20 controls (C). At T0, circulating miRNA-195-5p and -451a were significantly upregulated in both AIS and TIA patients, compared to C. We also observed a progressive reduction of circulating miRNA levels at 24 h and 48 h in both AIS and TIA patients. Hypoxia inducible factor 1alpha (HIF-1α) serum level was significantly increased at T0, in both AIS and TIA patients, in comparison to C (both *p* < 0.01 vs. C) and it decreased in both AIS and TIA patients at 24 h and at 48 h, in comparison to T0 (both *p* < 0.01 vs. T0). Vascular endothelial growth factor (VEGF) serum level was significantly decreased at T0, in both AIS and TIA patients, if compared to C (both *p* < 0.01 vs. C) and increased, in both AIS and TIA patients, at 24 h and 48 h, if compared to T0 (both *p* < 0.01 vs. T0). The elevated expression of miRNA-195-5p and miRNA-451a significantly decreased over time at 24 h and 48 h, and it is associated with decreased HIF-α levels and increased VEGF serum levels. These data may suggest a role for this miRNAs as biomarker in the pathogenesis and prognosis of AIS patients and for the first time also in TIA patients.

## 1. Introduction

Acute ischemic stroke (AIS) is one of the main causes of mortality and morbidity worldwide [1]. It is characterized by an acute phase of cellular damage within hours after the ischemic insult, followed by a chronic phase of limited plasticity and regeneration [2,3]. The stroke pathophysiology in the acute phase involves oxidative stress, inflammatory response, mitochondrial dysfunction, energy failure, activation of glial cells, disruption of the blood–brain barrier (BBB) and changes in microRNA (miRNAs) expression [4]. These endogenous noncoding short single-stranded RNAs have recently emerged as essential gene regulators for the orchestration of brain development and function [5,6,7,8,9,10,11]. In particular, previous studies have indicated that miRNA expression may be related to the progression of ischemia in the cerebral artery [12]. In fact, the miRNAs were correlated with pathologies such as cerebral ischemia, tumor angiogenesis and atherosclerosis [5,6,12,13,14]. Furthermore, a recent study found a significant association between miRNA single nucleotide polymorphisms and the risk of ischemic stroke [15]. Lastly, evidence has shown the involvement of several miRNAs in post-ischemic angiogenesis, a crucial process to restore blood supply to ischemic regions after stroke [16]. Indeed, multiple Hypoxia-inducible factor 1alpha (HIF-1α)-induced miRNAs, called hypoxia-regulated microRNAs (HRMs), have been shown to modulate post-stroke angiogenesis by targeting the vascular endothelial growth factor A (VEGF-A) pathway [17,18,19,20,21]. Among them, the HRMs miR-195-5p has been reported to regulate negatively VEGF-A expression, by reducing the new vessel formation in the brain after a stroke [22]. Particularly, miR451a seems to be correlated to the angiogenesis occurring in cancer development [23,24,25,26,27,28]. However, it has never been correlated to the angiogenesis of ischemic stroke. Since this process is crucial, not only to restore the blood flow to the ischemic area, but also to promote neurogenesis and to improve neurological functions [29], miR-195-5p and miR-451a could be an important therapeutic target for AIS.

As they are detectable and stable in many bodily fluids, including blood, miRNAs can serve also as diagnostic stroke biomarkers in humans [30,31,32,33]. Up to now, no study has investigated on the expressions of upregulated circulating miR-195-5p and miR-451a in serum of patients with AIS. In addition, no study has previously compared circulating miRNAs levels in AIS with those of patients affected by transient ischemic attack (TIA). Thus, in this observational study we evaluate the expression of circulating miR-195-5p and miR-451a in both AIS and TIA patients in comparison with control subjects, in order to verify if they could be valuable biomarkers for rapid diagnosis and prognosis of ischemic stroke, as well as promising therapeutic agents.

## 2. Materials and Methods

### 2.1. Patients Enrollment and Inclusion Criteria

The study population included 18 patients with TIA, 18 patients with AIS (confirmed by neuroimaging) and 20 patients without a history of ischemic stroke (control group, C) (Table 1). AIS was defined as an episode of acute neurological dysfunction caused by focal cerebral ischemia, based on objective imaging techniques such as computed tomography scan (CT) or magnetic resonance imaging scan (MRI) and clinical evidence of cerebral focal ischemic injury based on symptoms of any duration. TIA was defined as a transient episode of neurological dysfunction (that disappeared within 24 h) caused by focal brain, spinal cord, or retinal ischemia, without acute infarction [34]. Blood samples were obtained from patients at admission and then at 24 and 48 h after hospital admission. The diagnosis was confirmed with medical imaging diagnostic methods (CT and MRI).

Inclusion criteria were the following: age >60 years, APACHE II score evaluation <22, Cincinnati Score positive for neurological symptoms at admission (dysarthria, hemiparesis etc.) and neuroimaging positive [35] and signed informed consent. Patients in the control group had no history of cerebrovascular diseases. 

Exclusion criteria were: body temperature > 37.5 °C, history of surgery in the past 6 months, acute coronary disease, acute arrhythmias, severe anemia (Hgb < 7.5 g/dL), history of cancer, hemorrhagic stroke and participation in other clinical studies. All patients or one of their family members gave informed consent. The Ethical Review Board of North Campania approved the study (CECN/802).

### 2.2. MiRNA Isolation and Quantitative Reverse Transcription PCR (qRT-PCR) Analysis

Total RNA was isolated using QIAZOL (Qiagen, Milan, Italy) and Mirneasy serum/plasma kit (Qiagen miRNeasy Mini Kit, Milan, Italy) according to the manufacturer’s instructions. For each RNA extraction, 250 mL of once-thawed plasma was added to 750 mL QIAZOL (Qiagen, Milan, Italy). The solution was vortexed at high speed for 15 s and incubated at room temperature for 5 min. Synthetic sequences of Syn-cel-miR-39-3p miScripit miRNA Mimic 5 nM (Qiagen, Milan, Italy) were added to monitor the efficiency of miRNA isolation, followed by 200 µL of chloroform. Each tube was vortexed vigorously for 30 s and then was left at room temperature for 5 min. Phase separation was achieved by centrifuging the sample at 12,000 g for 20 min at 4 °C. After centrifugation, 350 µL of the aqueous phase was carefully transferred to a new tube for spin column purification. RNA was eluted in nuclease-free water by passing a few times through a pipette tip. RNA quality and quantity were measured by using Nanodrop spectrophotometer (Nanodrop 2000c Spectrophotometer, Thermo Fisher Scientific, Milan, Italy) and RNA integrity was determined by gel electrophoresis. A total of 600 ng of RNA were used for cDNA synthesis from mature miRNAs, following the miScript II RT Kit (Qiagen, Milan, Italy), using the miScript Reverse Transcriptase Mix, 10 miScript Nucleics Mix, and 5 miScript HiSpec Buffer. The mixture was incubated for 60 min at 37 °C and for 5 min at 95 °C to inactivate miScript Reverse transcriptase mix. This cDNA placed on ice was diluted in RNase free water (20 µL of cDNA, previously obtained as above, mixed with 180 µL of water). miR-195 (MIMAT0000461, miRBase), miR-451 (MIMAT000163, miRBase) and miR-39 (MIMAT0000010, miRBase) profiling was performed using specific MiScript Primer Assays (for has-miR-195-5p MS00003703; for has-miR-451a MS00004242; for cel-miR-39-3p MS00019789 Qiagen, Milan, Italy) and MiScript SYBR Green PCR Master Mix (Qiagen, Milan, Italy), by incubating the samples at 95 °C for 15 min; 40 cycles of 94 °C for 15 s; 55 °C for 30 s, and 70 °C for 30 s. The analysis of the miRNA expression levels was carried out on a CFX96 Touch TM Real-Time PCR Detection System (Biorad Laboratories Srl, Milan, Italy).

Data analysis of miRNA was performed using the ΔΔCT-method of relative quantization, according Marfella et al. [36] Syn-cel-miR-39-3p was used for normalization of qRT-PCR results. Δ*C*t value for miR-195 and miR-451 was calculated using the formula Δ*C*t = Ct miRNA − Ct Syn-cel-miR-39-3p. ΔΔ*C*t for each miRNA across the different group was calculated using the formula: ΔΔ*C*t = Δ*C*t of AIS or TIA group − Δ*C*t of control group. Expression fold change was obtained as 2^^−ΔΔ*C*t^, the normalized gene expression (2^^−Δ*C*t^) in AIS or TIA group divided the normalized gene expression (2^^−Δ*C*t^) in the control group. Expression fold change data are reported as fold regulation values, equal to the fold change values for fold change > 1 (upregulation) or expressed as the negative inverse of the fold change for fold change values < 1 (downregulation).

HIF-1α and VEGF-A serum levels were expressed as mean ± S.E.M of the observations (*N* = 12 in C group; *N* = 18 in AIS and TIA groups). 

### 2.3. Serum HIF-1α and VEGF-A ELISA Assays

Serum HIF-1α and VEGF-A levels were measured using respectively the HIF1A Human ELISA Kit (EHIF1A Thermo Fisher Scientific, Milan, Italy) and the VEGF-A Human ELISA Kit (BMS277-2 Thermo Fisher Scientific, Milan, Italy) according the manufacturer’s protocols.

### 2.4. Statistical Analysis

Statistical analyses were assessed either by Student’s 𝑡-test (when only two groups were compared) or by two-way analyses of variance (ANOVA), followed by Dunnett’s *post hoc* test (more than two experimental groups). Particularly, for miRNA analysis, p values were calculated based on Student’s 𝑡-test of the replicate 2^^−Δ*C*t^ values ± the standard error of the mean (S.E.M.) for each miRNA in control, AIS or TIA groups (*N* = 12 in C group; *N* = 18 in AIS and TIA groups). 

Three independent experiments were performed for both qRT-PCR and ELISA determinations. For all the results, a probability of *p* < 0.05 was considered sufficient to reject the null hypothesis. The criteria of differential expression were *p* < 0.05 and *p* < 0.01. 

## 3. Results

The clinical characteristics are reported in Table 1.

### 3.1. MiRNAs Timing in AIS and TIA Patients 

**Figure 1:**
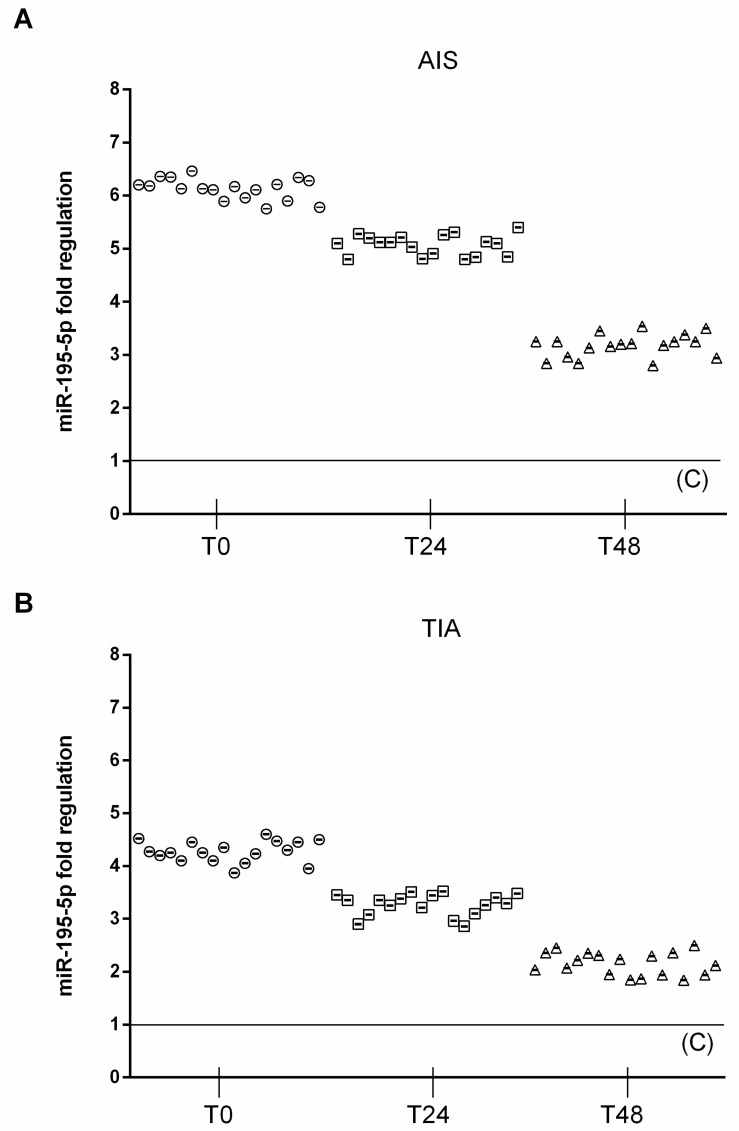
Expression levels of miRNA 195-5p. (**A**) Scatter plots of miR-195-5p as fold regulations in AIS patients (*N* = 18); control value reported as 1. (**B**) Scatter plots of miR-195-5p as fold regulations in TIA patients (*N* = 18); control value reported as 1.

At admission (T0), circulating miRNA-195-5p (2^^−Δ*C*t^ = 5.5 ± 0.4) were significantly upregulated in AIS patients, compared to both TIA (2^^−Δ*C*t^ = 3.9 ± 0.35, *p* < 0.01 vs. TIA) and control subjects (2^^−Δ*C*t^ = 0.91 ± 0.1, *p* < 0.01 vs. C). In AIS patients, at 24 h after admission, circulating miRNA-195-5p (2^^−Δ*C*t^ = 4.6 ± 0.3) was significantly upregulated, compared to TIA (2^^−Δ*C*t^ = 3.1 ± 0.4, *p* < 0.01 vs. TIA) and control subjects (2^^−Δ*C*t^ = 0.83 ± 0.1, *p* < 0.01 vs. C). In AIS patients, at 48 h after admission, circulating miRNA-195-5p (2^^−Δ*C*t^ = 2.9 ± 0.28) was significantly upregulated, compared to TIA (2^^−Δ*C*t^ = 1.9 ± 0.35, *p* < 0.01 vs. TIA) and control subjects (2^^−Δ*C*t^ = 0.88 ± 0.1, *p* < 0.01 vs. C) (Figure 1). 

**Figure 2:**
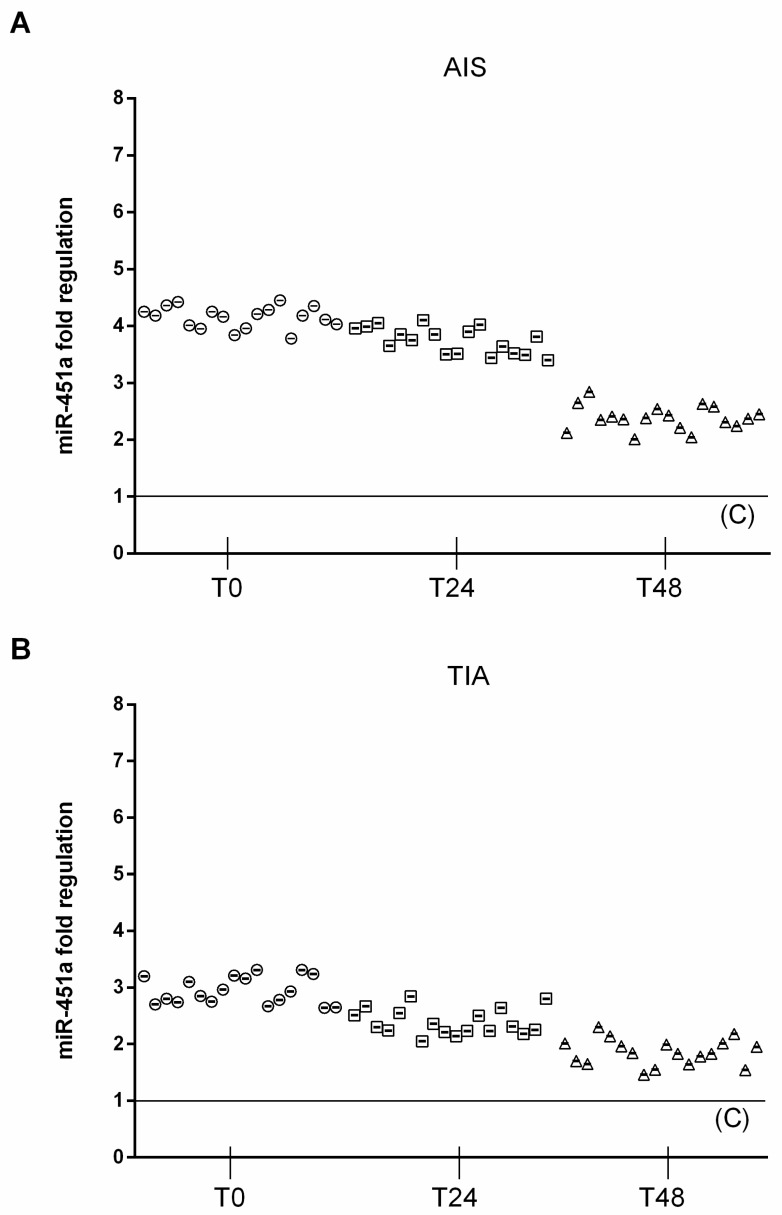
Expression levels of miRNA 451a. (A) Scatter plots of miR-451a as fold regulations in AIS patients (*N* = 18); control value reported as 1. (B) Scatter plots of miR-451a as fold regulations in TIA patients (*N* = 18); control value reported as 1.

Similarly, circulating miRNA-451a (2^^−Δ*C*t^ = 9.26 ± 0.82) was significantly upregulated in AIS patients at T0 compared to both TIA (2^^−Δ*C*t^ = 6.5 ± 0.52, *p* < 0.01 vs TIA) and control subjects (2^^−Δ*C*t^ = 2.2 ± 0.5, *p* < 0.01 vs. C). In AIS patients, 24 h after admission, circulating miRNA-451a (2^^−Δ*C*t^ = 8.2 ± 0.66) was significantly upregulated, compared to TIA (2^^−Δ*C*t^= 5.2 ± 0.63, *p* < 0.01 vs. TIA) and control subjects (2^^−Δ*C*t^ = 2.0 ± 0.5, *p* < 0.01 vs. C). In AIS patients, 48 h after admission, circulating miRNA-451a (2^^−Δ*C*t^ = 5.3 ± 0.55) was significantly upregulated, compared to TIA (2^^−Δ*C*t^ = 4 ± 0.48, *p* < 0.01 vs. TIA) and control subjects (2^^−Δ*C*t^ = 2.3 ± 0.5, *p* < 0.01 vs. C) (Figure 2).

### 3.2. HIF-1α in AIS and TIA Patients

**Figure 3:**
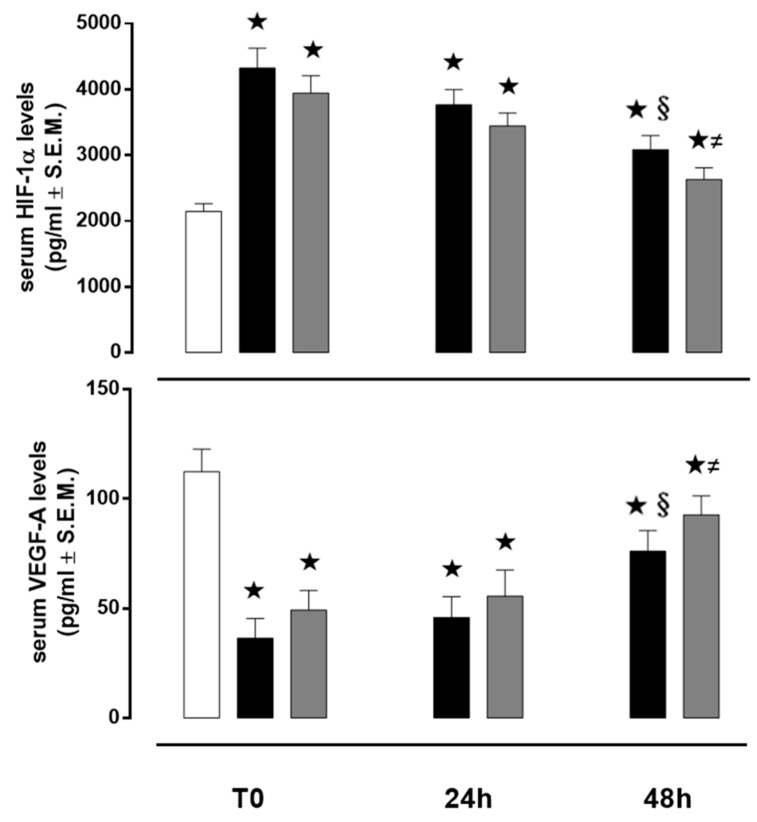
Serum levels of HIF-1α and VEGF-A. Values are expressed as pg/mL ± S.E.M. The control group is reported as white column; AIS group is reported as black column and TIA group is reported as grey column. ★ *p* < 0.01 vs. C; **§** *p* < 0.05 vs. AIS T0; ≠ *p* < 0.05 vs. TIA T0.

At T0, HIF-1α serum level, in control subjects was 2145 ± 116 pg/mL and it was significantly increased in both AIS and TIA patients (4325 ± 300 and 3943 ± 265 pg/mL, respectively; both *p* < 0.01 vs. C). At 24 h, HIF-1α serum level, in control subjects, was 2205 ± 118 pg/mL and it significantly decreased, in comparison to T0, in both AIS and TIA patients (3765 ± 231 and 3445 ± 197 pg/mL, respectively, both *p* < 0.01 vs. T0). At 48 h, HIF-1α serum level, in control subjects, was 2095 ± 115 pg/mL and it significantly decreased, in comparison to T0, in both AIS and TIA patients (3087 ± 213 and 2632 ± 175 pg/mL, respectively, both *p* < 0.01 vs. T0) (Figure 3). 

### 3.3. VEGF in AIS and TIA Patients 

VEGF serum level, in control subjects was 112 ± 10 pg/ mL, and it was significantly decreased, at T0, in both AIS and TIA patients (36 ± 8 and 49 ± 8 pg/ mL, respectively; both *p* < 0.01 vs. C). At 24 h, VEGF serum level, in control subjects was 116 ± 11 pg/ mL and it significantly increased, in comparison to T0, in both AIS and TIA patients (45 ± 9 and 55 ± 12 pg/mL, respectively). Similarly, at 48 h, VEGF serum level, in control subjects, was 115 ± 12 pg/mL and it significantly increased, in comparison to T0, in both AIS and TIA patients (76 ± 9 and 92 ± 8 pg/mL, respectively, both *p* < 0.01 vs. T0) (Figure 3).

## 4. Discussion

Traditionally, the diagnosis of stroke is mainly dependent upon examination by a clinical care provider, and various neuroimaging techniques [37]. Therefore, there is great need for a reliable and easily detectable circulating biomarker for both transient and acute ischemic stroke risk. The role of miRNA in patients with ischemic stroke is a developing field, with growing interest for their potential application as biomarker for rapid diagnosis and prognosis of ischemic stroke, as well as therapeutic agents [38]. Patient-based studies have already reported some alterations in circulatory miRNA expression during cerebral ischemia [39]. In fact, it has been reported that miRNAs regulate a number of genes and pathways associated with ischemic stroke including coagulation, platelets and thrombus formation [40]. In this regard, previous studies have demonstrated that a number of circulating miRNAs associated with deregulation of neurovascular integrity during a stroke mediated inflammation [41]. In particular, it has been recently reported that the overexpression of miR-34a-5p in the circulatory system of patients suggests the induction of brain cell apoptosis during acute ischemic stroke [42].

Although it is well known that activation of VEGF-A mediated by (HIF-1α)-induced miRNAs (HRMs) plays a key role in cerebral ischemia, and the HMRs miR-195-5p and miR-451a have been shown to target VEGF-A factor in several experimental settings [23,24,25,26,27,28], no clinical study investigated the exact correlation between their expression, the HIF-1 expression and the outcomes of AIS and TIA patients. In the present study we reported for the first time elevated circulating miRNAs (-195-5p and -451a) in AIS and TIA patients, compared to control subjects. We also reported that a significant increase of HIF-1α and a significant decrease of VEFG-A expression levels are both present in AIS and TIA patients at the time of the ischemic event, in comparison to control subjects.

To this regard, the initial 48 h post-ischemic period was characterized by a trend of an increasing VEGF-A serum concentration, suggesting that VEGF-A may mediate endothelial progenitor cells proliferation in the early phase of an ischemic stroke [43]. This increase was associated with a significant reduction in miR-195-5p and miR-451a serum levels 48 h after ischemic event, further emphasizing the importance of these miRNAs in the regulation of blood flow restoration in the ischemic area. Interestingly, both miR-195-5p and miR-451a levels were significantly upregulated in AIS patients compared to TIA group. This elevation could be related to the higher severity of the ischemic event during AIS, especially when associated with lower VEGF-A serum levels. These were detected in AIS patients 48 h after cerebral ischemia, indicating a reduction in brain angiogenesis in the AIS group compared to TIA patients. To our knowledge, this data is reported for the first time.

The importance of this study also resides in the temporal pattern of miR-195-5p and miR-451 changes as biomarker of stroke. Noteworthy, after cerebral ischemia, there are two major events occurring at levels of vessels: the improvement of the collateral flow during an early phase and then the proper angiogenesis, requiring time and occurring in the late phase [18]. Although we cannot discern at the moment between them, the temporal change of these two miRNAs may account for the VEGF-A mediated late development of new collateral vessels into the brain in order to balance the blood flow. Therefore, they may account for sign of an initial good cerebral tissue outcome following stroke (e.g., local recruitment of endothelial progenitor cells).

Despite this evidence, this study does not further discuss why the expected positive correlation between HIF-1α alpha and VEGF-A resulted here in a negative correlation. It is our opinion that this may probably be due to the fact that HIF-1α could be a trigger when hypoxia occurs, however as the stroke processes versus the re-oxygenation its values decreases due to the reoxygenation of the tissue. In contrast, the HIF-induced synthesis of VEGF-A while being very low and superimposable to the HIF-1α expression increases in time depending manner due to presence of oxygen, as it is due. Globally, still significant high levels of HIF-1α with respect to the patients without history of ischemic stroke are evident through the study at the different time point considered.

Thus, circulating levels of miR-195-5p and miR-451a may have implications for the development of a desirable biomarker and therapy for TIA and AIS. A large study in the future may be helpful to elucidate the utility of these important biomarkers in clinical practice.

## 5. Limitations

Our data were collected in a small group of patients and at a single institution and cannot be generalized to other patient groups. 

## Figures and Tables

**Table 1:** Clinical characteristics in control, Acute ischemic stroke (AIS) and Transient Ischemic attack (TIA) groups. BMI (Body Mass Index), Systolic Blood Pressure (SBP), Diastolic Blood Pressure (DBP). The values are indicated by percentage and mean ± SE.

	Control	AIS	TIA	*p* Value
*N* (M)	20 (11)	18 (6)	18 (7)	
Age (years)	72.7 ± 3	73.3 ± 2	74.2 ± 3	n.s.
BMI (kg/m^2^)	26.4 ± 3	27.2 ± 2	25 ± 3	n.s.
SBP (mmHg)	138 ± 6	145 ± 6	142 ± 7	n.s.
DBP (mmHg)	81 ± 3	83 ± 2	85 ± 2	n.s.
Hypertension (%)	6 (50)	10 (55)	9 (50)	n.s.
Diabetes (%)	4 (33)	8 (44)	6 (33)	n.s.
Smoking (%)	3 (25)	5 (27)	7 (38)	n.s.
Hyperlipidemia (%)	4 (33)	8 (44)	7 (38)	n.s.
